# Cost-Effectiveness in Critical Care: A Systematic Review of Empirical Evaluations

**DOI:** 10.3390/healthcare13212783

**Published:** 2025-11-03

**Authors:** Fotios Tatsis, Mary Gouva, Elena Dragioti, Foteini Veroniki, Konstantinos Stamatis, Georgios Papathanakos, Vasilios Koulouras

**Affiliations:** 1 Intensive Care Unit, University Hospital of Ioannina, 45500 Ioannina, Greece; f.tatsis@uoi.gr (F.T.); fenidio@yahoo.gr (F.V.); kdstamatis@gmail.com (K.S.); gppthan@icloud.com (G.P.); 2 Scientific Laboratory of Psychology and Person-Centered Care, Department of Nursing, School of Health Sciences, University of Ioannina, 45500 Ioannina, Greece; gouva@uoi.gr (M.G.); dragioti@uoi.gr (E.D.)

**Keywords:** critical care, cost-effectiveness, economic evaluation, cost, ICU, ICER

## Abstract

**Background**: Intensive Care Units (ICUs) provide essential therapies but are among the most resource-intensive areas of healthcare. Rising demand and escalating costs highlight the need for robust cost-effectiveness analyses (CEAs) to support efficient resource allocation. This review systematically synthesizes the available economic evaluations of ICU interventions and, where feasible, conducts meta-analyses to assess their value and inform policy, clinical decision-making, and future research. **Methods**: A systematic review and meta-analysis were conducted following PRISMA guidelines, registered in PROSPERO (CRD420251130870). Eligible studies were trial-based economic evaluations in adult ICU populations, reporting cost-effectiveness outcomes such as cost per life-year gained, life saved, or adverse event avoided. A comprehensive search was performed in PubMed, Scopus, and Web of Science, with data extracted independently by two reviewers. Costs were standardized to 2024 USD. Pooled estimates were synthesized using the Incremental Net Benefit (INB) framework. **Results**: From 5003 records, 15 trial-based economic evaluations met the inclusion criteria. Studies spanned diverse regions and ICU populations, assessing pharmacological, preventive, and organizational interventions. Reported ICERs ranged from $6904 to $69,346 per life-year gained and $51,664 to $476,499 per life saved, with several preventive and protocol-based strategies found to be dominant. Eight studies contributed to the meta-analysis, yielding a pooled INB of $15,123. **Conclusions**: This review highlights the wide variability in cost-effectiveness of ICU interventions, with preventive and quality-improvement strategies most often found to be economically dominant. Pharmacological and life-support therapies showed inconsistent value, underscoring the need for context-specific appraisal. Future evaluations should adopt standardized reporting and real-world data to better inform critical care policy and resource allocation.

## 1. Introduction

Intensive Care Units (ICUs) are essential for the management of critically ill patients, offering advanced monitoring and life-sustaining therapies that are often unavailable elsewhere in the healthcare system. However, the delivery of such high-level care comes at a substantial economic cost. ICUs are consistently among the most expensive components of hospital-based care, accounting for up to 20% of total hospital expenditures while serving a relatively small proportion of patients [1,2]. Daily costs per ICU bed are markedly higher than those of general ward beds, driven by labor intensity, advanced medical technologies, and prolonged lengths of stay [3]. The average daily ICU cost is estimated at US$3000–4000 in high-income countries and US$500–1000 in middle-income regions [4]. In the United States, critical care consumes nearly 1% of GDP and 13% of hospital spending [5]. These expenditures are expected to rise further due to aging populations, the increasing prevalence of chronic diseases, and the growing demand for intensive care services. ICU bed availability varies widely: about 20–30 per 100,000 people in North America and Western Europe, versus fewer than 5 in many low- and middle-income countries, and under 1 per 100,000 in parts of sub-Saharan Africa [6,7]. ICU mortality remains 10–20%, reaching >35% for severe sepsis and multi-organ failure [8]. As populations age and comorbidities rise, global demand for ICU care continues to grow—highlighting the need for cost-effectiveness frameworks to ensure equitable and sustainable resource allocation [9].

In this context, the need for rigorous economic evaluations—particularly cost-effectiveness analyses (CEAs)—has become more pressing than ever. CEAs aim to inform resource allocation decisions by quantifying the ratio of costs to health outcomes, typically measured as life-years gained or life saved. Despite the methodological maturity of economic evaluation in other areas of medicine, its application in ICU settings remains relatively underdeveloped [10,11,12]. A number of barriers contribute to this gap, including the heterogeneity of ICU populations, the acute and unpredictable nature of critical illness, and the ethical and practical difficulties in conducting randomized controlled trials in this setting [13].

Moreover, synthesizing economic evidence in ICU settings through systematic reviews and meta-analyses poses significant methodological challenges. First, the available studies often vary widely in terms of design, perspective, time horizon, cost components, and currencies, introducing substantial heterogeneity [14,15]. Second, many economic evaluations in intensive care rely on decision-analytic models, which are highly sensitive to assumptions and parameter inputs, limiting their comparability. Third, outcome measures differ substantially, with some studies reporting incremental cost-effectiveness ratios (ICERs), others presenting cost per life-year gained, and still others focusing on net monetary benefit or return on investment, making pooled estimates challenging and, in some cases, inappropriate [16].

Furthermore, the quality of reporting in ICU CEAs is variable, with some studies failing to disclose key methodological details such as sensitivity analyses, justification for chosen time horizons, or the inclusion of indirect costs. These inconsistencies highlight the importance of standardized reporting frameworks, such as the CHEERS guidelines, to improve the transparency and comparability of future economic evaluations [17]. Recent reviews have attempted to map the landscape of health economic evaluations in the ICU, including cost-effectiveness, cost-utility, and cost-minimization studies, incorporating both trial-based analyses and simulation models [10,12]. Although previous reviews have explored economic evaluations in intensive care, they primarily included model-based analyses or mixed trial and simulation data, without focusing exclusively on empirical, patient-level evaluations. This highlights a persisting gap in synthesizing real-world, empirically derived cost-effectiveness evidence. Therefore, the present review aims to address this gap by systematically identifying, assessing, and pooling data from trial-based economic evaluations to provide robust estimates of incremental net benefit and to guide policy and clinical decision-making.

Given these challenges, this study undertakes a systematic review of the published cost-effectiveness literature pertaining to ICU interventions, with the goal of mapping the current evidence base, assessing methodological quality, and identifying gaps for future research. In addition, a meta-analysis will be conducted where feasible, focusing on interventions for which comparable outcome and cost data are available. Therefore, this review seeks to provide a comprehensive synthesis of the economic value of ICU therapies and inform health policy, clinical decision-making, and research prioritization in the context of constrained healthcare resources.

## 2. Materials and Methods

### 2.1. Study Design and Registration

This systematic review and meta-analysis were conducted according to the methodological principles outlined in the Cochrane Handbook for Systematic Reviews of Interventions and the Center for Reviews and Dissemination (CRD) guidance, ensuring a transparent and reproducible process for study identification, selection, data extraction, and synthesis. The reporting of results followed the Preferred Reporting Items for Systematic Reviews and Meta-Analyses (PRISMA) statement. The objective was to evaluate the cost-effectiveness of health interventions in ICU settings using studies based on primary data, excluding model-based or simulation-based economic evaluations. We focused on studies that reported incremental cost-effectiveness ratios (ICERs) in terms of alternative clinical outcomes such as cost per life-year gained, cost per life saved, or cost per adverse event avoided.

Eligible studies included prospective or retrospective cost-effectiveness studies, embedded within randomized controlled trials, observational cohorts, or before-and-after interventional designs, as long as they used real-world, patient-level data to assess both costs and outcomes. Two independent reviewers screened studies using the predefined eligibility criteria based on the Cochrane framework. Data extraction followed standardized templates consistent with CRD guidance, and discrepancies were resolved through consensus. Meta-analysis was planned using the Incremental Net Benefit (INB) framework with a fixed willingness-to-pay (WTP) threshold.

The review protocol was developed a priori, and the data extraction sheet was piloted and iteratively updated. The protocol for this study is registered in PROSPERO with registration number: CRD420251130870. No major deviations from the original plan occurred.

### 2.2. Framework for Review Question Formulation

To ensure methodological clarity and transparency, the formulation of the review question in this study was guided by the PICO framework, which is widely used in clinical and health economic research for structuring systematic reviews. Specifically, the population of interest included adult patients admitted to Intensive Care Units with critical illness, such as sepsis, acute kidney injury, ventilator-associated pneumonia, and other life-threatening conditions commonly encountered in intensive care settings. The interventions assessed were clinical, diagnostic, or preventive strategies implemented within the ICU context, including pharmacologic agents (e.g., drotrecogin alfa), monitoring technologies (e.g., pulmonary artery catheterization), infection control measures (e.g., selective digestive decontamination, hand hygiene protocols), and diagnostic tools (e.g., contrast-enhanced transthoracic echocardiography). Comparators included either standard care or alternative interventions, provided that the study offered a comparative analysis of both costs and outcomes. “Standard care” was defined according to the locally adopted routine clinical practice or guideline-based management described in each original study, acknowledging that such care may differ across countries and institutions. Eligible outcomes focused on economic endpoints derived from trial-based evaluations. These included incremental cost-effectiveness ratios (ICERs) expressed as cost per life-year gained, cost per life saved, cost per adverse event avoided, or cost per quality-adjusted life-year (QALY). Where data permitted, incremental net benefit (INB) estimates were also calculated or extracted to allow pooling of cost-effectiveness evidence across studies. Measures of uncertainty, such as standard errors or confidence intervals, were recorded to facilitate quantitative synthesis.

### 2.3. Eligibility Criteria

We included full economic evaluations that assessed the cost-effectiveness or cost-minimization of clinical or diagnostic interventions using primary, patient-level data. The study population included adult critically ill patients admitted to Intensive Care Units (ICUs), and eligible studies were required to report both cost and clinical effectiveness outcomes. Studies were included if they were conducted alongside randomized controlled trials or observational designs and if they compared an intervention implemented in ICU settings against standard care or an alternative ICU intervention.

Economic outcomes of interest encompassed incremental cost-effectiveness ratios (ICERs), expressed as cost per life-year gained, cost per life saved, cost per quality-adjusted life-year (QALY), or incremental net benefit (INB). Studies conducted in hospital ICUs in any country and published in English between 1995 and 2025 were considered eligible.

Studies were excluded if they relied solely on decision-analytic or simulation models, such as Markov or microsimulation approaches, or if they presented partial economic evaluations reporting only costs without effectiveness data or a comparator. We also excluded studies focusing exclusively on pediatric populations, non-ICU settings (e.g., emergency or intermediate care units), or theoretical and narrative reports without empirical data. Furthermore, cost-utility analyses reporting only QALYs without alternative clinical outcome-based ICERs were not eligible.

### 2.4. Search Strategy

A comprehensive literature search was conducted in three major databases: PubMed, Scopus, and Web of Science. The search included studies published between 1 January 1995, and 31 July 2025. We used combinations of keywords and controlled vocabulary (e.g., MeSH terms) related to economic evaluation, cost-effectiveness, and critical care. The search query consisted of the following terms: (*cost-effectiveness* OR *economic evaluation*) AND (*intensive care unit* OR *critically ill patients*).

The search was designed to identify studies that reported full economic evaluations using primary data in ICU populations. No language restrictions were applied during the database search or initial screening, in line with the registered protocol. However, during full-text assessment, only studies published in English met the inclusion criteria and provided sufficient methodological and economic detail for extraction. This approach reflects feasibility rather than a predefined language limitation.

The reference lists of all included studies and relevant reviews were also manually screened to identify any additional eligible studies; however, this process did not yield any further eligible records beyond those retrieved through database searches. Gray literature (e.g., conference proceedings, theses, or non-peer-reviewed reports) was not systematically searched, as the inclusion criteria required full economic evaluations with complete methodological and cost data, which are typically unavailable in such sources. This approach was consistent with the predefined protocol and ensured methodological transparency and reproducibility.

Two reviewers independently and blindly screened all titles and abstracts for relevance, followed by an independent full-text assessment of potentially eligible studies. Reference management and de-duplication were performed using Mendeley. Discrepancies between reviewers were resolved through discussion, and a third reviewer was consulted in cases of persistent disagreement.

### 2.5. Data Extraction and Processing

A standardized data extraction form was developed a priori and piloted on a subset of included studies. Two reviewers independently extracted relevant data, with discrepancies resolved through discussion and, when necessary, consultation with a third reviewer.

For each study, we extracted key characteristics including: authorship, year of publication, country, setting, population sample, intervention and comparator, time horizon, perspective, currency and base year, and outcome type.

Economic results were extracted or calculated, including the Incremental Cost (ΔC) and the Incremental Effectiveness (ΔE), expressed in life-years gained, lives saved, or adverse outcomes avoided. We also recorded the Incremental Cost-Effectiveness Ratio (ICER) and its unit. Where available, 95% confidence intervals (CIs) or standard errors (SEs) for ΔC and ΔE were also extracted.

If ΔCost and ΔEffect were reported without variance measures, we estimated standard errors or confidence intervals using reported group-level statistics or plausible assumptions based on the study’s design, consistent with recommendations in the health economic literature [16,18], or we reconstructed approximate variances from published cost-effectiveness acceptability curves (CEACs) or from subgroup ICERs reported in the paper [19].

### 2.6. Assessment for Reporting Quality

The quality of reporting of the included economic evaluations was assessed using the Consolidated Health Economic Evaluation Reporting Standards (CHEERS) 2022 checklist [20], developed by the International Society for Pharmacoeconomics and Outcomes Research (ISPOR). This 28-item checklist provides structured guidance for evaluating the transparency and completeness of health economic studies across domains such as study context, perspective, comparators, outcome measures, analytical methods, uncertainty, and conflicts of interest.

Each included study was independently reviewed by two researchers, who evaluated adherence to individual CHEERS items. Items were rated as “fully reported,” “partially reported,” or “not reported.” Any discrepancies were resolved by consensus. The total number of fully reported items was calculated for each study to provide an overall estimate of reporting quality.

### 2.7. Currency Conversion and Cost Standardization

To enable comparability across studies conducted in different countries and years, all cost data were converted to 2024 US dollars (USD). For studies reporting costs in currencies other than USD or in earlier years, we adjusted values for inflation using the Purchasing Power Parity (PPP)-adjusted proposed on the cost converter tool from CCEMG-EPPI Center [21]. These transformations were applied consistently to all relevant cost components, such as ICERs and INB estimates, to facilitate meaningful synthesis across studies.

### 2.8. Data Synthesis and Meta-Analysis

We synthesized economic outcomes across studies using the Incremental Net Benefit (INB) approach, which allows for direct comparison and pooling of cost-effectiveness estimates. The INB for each study was calculated using the following formula:

INB = λ × ΔE − ΔC

where λ represents the willingness-to-pay threshold, set at $30,000, ΔE is the incremental effectiveness, and ΔC is the incremental cost. All monetary values were standardized to 2024 USD as described previously.

We conducted a random-effects meta-analysis of INB values. Heterogeneity was quantified using the I^2^ statistic. All analyses were conducted in Stata/SE, version 17.0 (Stata Corp., College Station, TX, USA).

## 3. Results

### 3.1. PRISMA Flow and Study Selection Results

**Figure 1:**
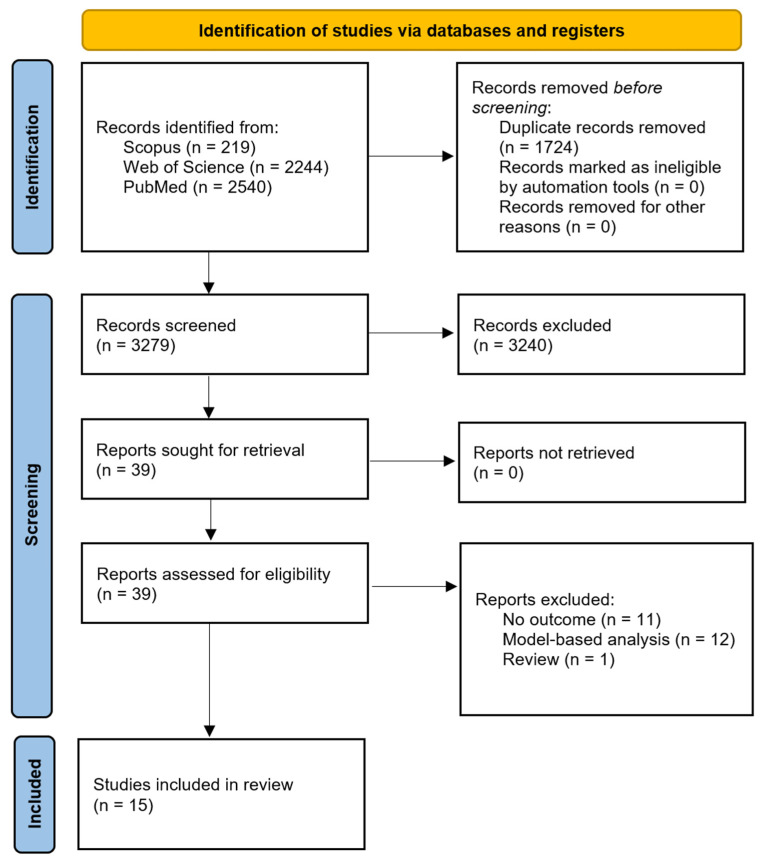
PRISMA flow diagram.

A total of 5003 records were initially retrieved through the electronic databases. After removing 1724 duplicates, 3240 records were excluded following title and abstract screening due to irrelevance or not meeting the eligibility criteria. As a result, 39 full-text articles were assessed for eligibility. Following a detailed evaluation, 15 studies met the inclusion criteria and were incorporated into the present systematic review and meta-analysis. The study selection process is presented in a PRISMA flow diagram (Figure 1).

### 3.2. Characteristics of Included Studies

Table 1 summarizes the general characteristics of the studies included in this systematic review. A total of 15 trial-based economic evaluations conducted in adult intensive care settings were included. The studies were carried out across diverse geographical regions: Europe (Italy, Sweden, France, Germany, the United Kingdom), North America (Canada, USA), South America (Brazil), Asia (Vietnam, Saudi Arabia), and Australasia (New Zealand, Australia). Sample sizes varied substantially, ranging from as few as 64 patients [22] to nearly 10,000 [23], reflecting both single-center analyses and large multi-center trials.

The patient populations were heterogeneous. Several studies focused on severe sepsis or septic shock [22,23,24,25,26,27], while others included mechanically ventilated patients [28,29], general ICU cohorts [30,31,32], or high-risk subgroups such as patients with central venous lines [33] or at risk of pressure ulcers [34]. Broader quality and safety interventions were also represented, including hand hygiene programs [35].

The interventions assessed were diverse, spanning pharmacological therapies (drotrecogin alfa, recombinant human activated protein C, angiotensin II, hydrocortisone), infection prevention strategies (probiotics, closed infusion containers, multilayer silicone dressings, hand hygiene), and organizational or protocol-driven approaches (quality improvement programs, sepsis management protocols, prolonged ICU care vs. early withdrawal of support, or monitoring with pulmonary artery catheters). Comparators were generally standard care, placebo, or historical controls.

The analytic perspectives varied, with most studies adopting a hospital or healthcare system perspective. The time horizons ranged from very short-term (e.g., 12 days) [34] to analyses extending over a lifetime [22,23,24,32,36]. Some studies reported results limited to the hospitalization episode or until discharge [29,33].

Overall, the 15 included studies provide a comprehensive and heterogeneous picture of economic evaluations in intensive care, covering different populations, interventions, and methodological approaches, thus offering valuable insights into the cost-effectiveness of ICU-based strategies in real-world settings.

**Table 1:** General characteristics of included studies.

Study	Country	Population	Setting	N	Intervention	Comparator	Perspective	Time Horizon
Berto, 2011 [22]	Italy	Severe sepsis/septic shock patients who had emergency surgery for intra-abdominal infection.	10 tertiary care ICUs	64	Polymyxin B hemoperfusion	Conventional therapy	Hospital	Lifetime
Tarricone, 2010 [33]	Italy	ICU patients with central lines	4 ICUs in an Italian teaching hospital	1446	Closed, fully collapsible plastic IV containers	Open glass infusion containers	Hospital	Hospital discharge
Heyland, 1998 [30]	Canada	General	12-bed, adult, medical–surgical ICU	690	Continue ICU care >14 days	Withdrawal of support after 14 days	Hospital	12 months
Manns, 2002 [24]	Canada	Severe sepsis	3 tertiary care hospitals with medical–surgical ICUs.	787	Drotrecogin alfa (activated)	Standard care	Healthcare	Lifetime
Busse, 2020 [36]	9 countries (North America, Australasia, and Europe)	Severe distributive shock	75 centers across 9 countries	321	Angiotensin II	Placebo	Healthcare	Lifetime
Ersson, 2018 [31]	Sweden	General ICU patients	12-bed mixed ICU in a 600-bed tertiary teaching hospital	5950	Quality improvement (QI) process	Historical cohort	Healthcare	7 years
Lau, 2022 [29]	3 countries (Canada/USA/Saudi Arabia)	Mechanically ventilated patients	44 ICUs	2650	Probiotics (Lactobacillus rhamnosus GG)	Placebo	Healthcare	Hospital discharge
Mayer, 2000 [28]	USA	Patients who are mechanically ventilated	N/A	510	Mechanical ventilation	Death	Healthcare	6 years
Dhainaut, 2007 [25]	France	Severe sepsis with multiple organ failure	85 ICUs	1096	Recombinant human activated protein C	Standard care	Hospital	NR
El Genedy, 2020 [34]	Germany	Patients at high risk for pressure ulcers	Seven ICUs	422	Multi-layered silicone foam dressing	Standard care	Hospital	12 days
Thu, 2015 [35]	Vietnam	ICU patients	2 main ICUs and 15 CCUs	984	Hand hygiene program	Before hand hygiene program	Hospital	NR
Riou Franca, 2006 [23]	France	Severe sepsis with multiple organ failure	ICU	9848	Drotrecogin alfa (activated)	Placebo	Healthcare	Lifetime
Thompson, 2022 [27]	New Zealand	Patients with septic shock	8 medical-surgical ICUs	419	Hydrocortisone infusion	Placebo	Healthcare	2 years
Stevens, 2005 [32]	Great Britain	Patients in the ICU	65 ICU	1014	Treatment without using a PAC	Routine PAC use	Healthcare	Lifetime
Assuncao, 2014 [26]	Brazil	Severe sepsis and septic shock.	1 ICU	414	Managed protocol	Usual care	Hospital	NR

NR = not reported.

### 3.3. Primary Outcomes

The 15 included studies reported primary cost-effectiveness outcomes, most commonly expressed as incremental cost per life-year gained (LYG), cost per life saved, or cost per adverse event prevented (e.g., CLABSI, HAI, pressure ulcers) (Table 2).

Among interventions targeting severe sepsis or septic shock, the incremental cost-effectiveness ratios (ICERs) were heterogeneous. Polymyxin B hemoperfusion in Italy was associated with a cost per LYG of $6904 [22], while drotrecogin alfa (activated) produced ICERs of $47,231 per LYG in Canada [24] and $19,664 per LYG in France [23]. Recombinant human activated protein C demonstrated an ICER of $36,042 per LYG [25]. By contrast, a managed sepsis protocol in Brazil was found to be dominant, achieving both improved outcomes and lower costs [26].

Studies evaluating critical care strategies also reported variable results. Prolonged ICU care beyond 14 days, compared to withdrawal of support, had an ICER of $7176 per LYG and $107,592 per life saved [30]. Mechanical ventilation in the USA was associated with a cost of $69,346 per LYG [28]. In contrast, hydrocortisone infusion in septic shock patients [27] was dominant.

Preventive and quality improvement interventions demonstrated more favorable cost-effectiveness profiles. A quality improvement process in Sweden [31], a hand hygiene program in Vietnam [35], and closed infusion containers in Italy [33] were all dominant, indicating cost savings with improved outcomes. Similarly, multilayered silicone foam dressings in Germany showed an ICER of $3091 per pressure ulcer avoided [34].

Other interventions yielded higher ICERs. Angiotensin II in distributive shock was associated with $10,756 per LYG [36]. In Lau et al. (2022), the mean hospital costs were high in both groups, consistent with the resource-intensive nature of critical care [29]. The average per-patient cost was $75,301 in the usual care group and $80,361 in the probiotic group, corresponding to an incremental cost of $4213. The difference in VAP incidence was small and statistically non-significant, leading to an incremental cost-effectiveness ratio of approximately $476,499 per life saved [29]. Avoidance of pulmonary artery catheters in the UK resulted in $51,664 per life saved [32].

Several interventions—particularly infection prevention programs and protocol-based approaches—were found to be dominant, while pharmacological therapies and life-support strategies displayed a wide range of ICERs, from highly cost-effective (<$10,000 per LYG) to very costly (>$400,000 per life saved).

Overall, across all studies, the ICERs for life-years gained ranged from $6904 to $69,346 per LYG, with several interventions reported as dominant. For cost per life saved, estimates varied widely, from $51,664 to $476,499, again with some studies showing dominance. For adverse event prevention, costs ranged from $3091 per event avoided to interventions that were dominant [33,35]. The results demonstrated considerable variability in cost-effectiveness estimates across studies, with preventive and protocol-based strategies more frequently reported as dominant than pharmacological interventions.

### 3.4. Secondary Outcome

Eight of the included studies reported incremental cost-effectiveness ratios (ICERs) in terms of cost per quality-adjusted life-year (QALY) (Table 3).

The estimates demonstrated wide variability across interventions and settings. Drotrecogin alfa (activated) was associated with $78,719 per QALY in Canada [24] and $32,772 per QALY in France [23], while recombinant human activated protein C yielded $60,070 per QALY in another French study [25]. Angiotensin II in distributive shock produced a comparatively favorable ICER of $15,700 per QALY across nine countries [36].

Results from ICU care strategies showed mixed findings. Mechanical ventilation in the USA was associated with a very high cost of $321,280 per QALY [28], whereas treatment without pulmonary artery catheter use in the UK resulted in $7059 per QALY [32]. A quality improvement process in Sweden was reported as dominant [31], while hydrocortisone infusion in New Zealand was found to be dominated, indicating higher costs with no additional benefit [27].

Overall, reported ICERs per QALY ranged from as low as $7059 per QALY [32] to as high as $321,280 per QALY [28]. Some interventions were found to be dominant [31], while others were dominated [27]. The results showed that several ICU interventions were associated with favorable cost-effectiveness estimates, although substantial variability was observed across therapies and contexts.

### 3.5. Results of Syntheses

Eight studies [22,23,24,25,27,28,30,36] were eligible for meta-analysis. Table 4 presents the incremental net benefit (INB) estimates and corresponding standard errors (SE) reported across the included studies.

The point estimates of INB varied substantially, ranging from a large negative value of $−67,675 [28] to a high positive value of $151,557 [30]. Several studies demonstrated positive INB estimates [22,23,27,36], whereas others reported negative values [24,25,28]. The associated SEs also showed wide variation, spanning from as low as 3687 [23] to as high as 79,626 [22], reflecting differences in sample size and study design.

**Figure 2:**
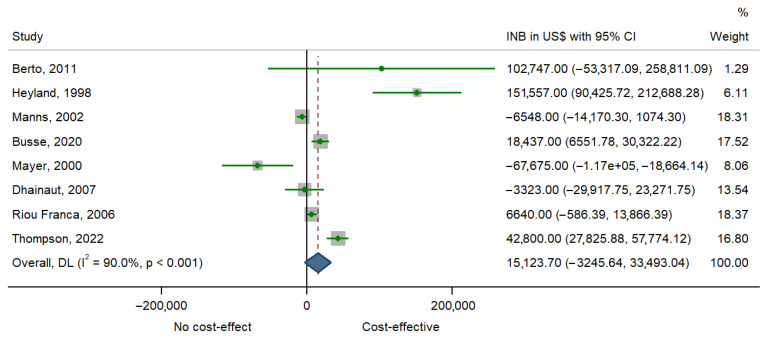
Forest plot of pooling INBs. The figure analyses eight studies. Concretely, it represents the mean difference of a study represented by squares and their 95% CIs represented by horizontal lines. The size of each square reflects the study weight. The diamond at the bottom represents the pooled mean difference, calculated using a Random Effects model [22,23,24,25,27,28,30,36].

The forest plot (Figure 2) displays the incremental net benefit (INB) estimates with their 95% confidence intervals for each study. Considerable heterogeneity is evident, as some studies reported positive INBs suggesting cost-effectiveness, while others indicated negative INBs. The pooled estimate using a random-effects model yielded an INB of US $15,123 (95% CI −3246 to 34,943), pointing towards cost-effectiveness but with substantial uncertainty. The high heterogeneity (I^2^ = 90%, *p* < 0.001) suggests that differences in study design, populations, and interventions strongly influence the observed outcomes. While the overall trend favors economic value, the wide variability limits the certainty of generalizable conclusions.

**Figure 3:**
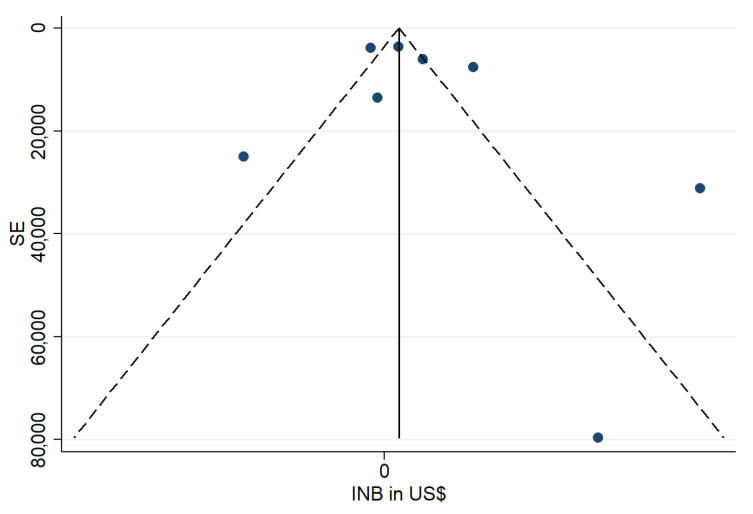
Funnel plot of pooling INB. Each blue dot represents an individual study included in the meta-analysis, plotted according to its Incremental Net Benefit (INB) on the x-axis and its standard error (SE) on the y-axis. The solid vertical line indicates the overall pooled INB estimate derived from the random-effects model. The diagonal dashed lines represent the pseudo 95% confidence limits within which studies are expected to lie in the absence of publication bias and with sampling variation only.

The funnel plot (Figure 3) was used to explore potential publication bias. Ideally, studies would be symmetrically distributed around the vertical line at INB = 0. In this case, the distribution appears somewhat asymmetric, with certain studies lying outside the expected region. This may reflect either true heterogeneity or potential publication bias.

### 3.6. Study Quality Assessment

The overall reporting quality of the included studies, as assessed using the CHEERS 2022 checklist [20], demonstrated considerable variability. Quality scores ranged from 54% to 94%, with an average score of 79%, corresponding to an overall classification of good reporting quality (Table A1). Five studies achieved excellent quality with scores equal to or above 85% [22,25,27,29,31]. Another seven studies were classified as very good (scores between 70% and 84%) [23,24,28,30,32,34,36]. Two studies demonstrated good quality with scores between 55% and 69% [33,35]. Finally, only one study was rated as having poor reporting quality (54%) [26].

Although most studies adequately reported the study population, comparators, cost measurements, and outcomes, several critical components were frequently missing. These included the absence of a pre-specified health economic analysis plan, limited reporting on distributional effects, and a near-total lack of stakeholder or patient engagement. Furthermore, justification for analytic assumptions and modeling approaches was inconsistently presented, even in studies that employed economic modeling.

These reporting gaps highlight the ongoing need for more comprehensive adherence to established health economic reporting standards. Greater consistency in methodological transparency, particularly regarding planning and stakeholder involvement, would strengthen the interpretability, reproducibility, and policy relevance of economic evaluations in ICU settings. The use of standardized frameworks such as CHEERS remains essential for improving both the quality and comparability of published cost-effectiveness research.

## 4. Discussion

This systematic review and meta-analysis synthesized current evidence from 15 economic evaluations conducted in intensive care unit (ICU) settings across diverse international contexts. The findings highlight substantial heterogeneity in cost-effectiveness across interventions, perspectives, and patient populations, underscoring the complexity of value-based decision-making in critical care. Three core themes emerge from this analysis: (1) the economic favorability of preventive and protocolized strategies, (2) the variability of pharmacological interventions, and (3) the methodological challenges in standardizing health economic evaluations in critical care.

### 4.1. Comparison with Existing Literature

Our findings reinforce trends identified in previous reviews. For instance, Ruiz-Ramos et al. (2017) demonstrated that the implementation of antimicrobial stewardship (AMS) programs in critical care can be cost-effective, with downstream reductions in infection-related morbidity and antibiotic use, supporting our findings on the economic dominance of interventions like hand hygiene and closed infusion systems [37].

Similarly, Møller et al. (2012) showed that the introduction of a ventilator care bundle in Danish ICUs was cost-saving and clinically effective in reducing ventilator-associated pneumonia (VAP), aligning closely with our observed economic benefit of quality improvement interventions in mechanically ventilated populations [38]. A more recent umbrella review by Zhu et al. (2024) also confirmed the efficacy of VAP prevention strategies, though cost-effectiveness outcomes were less frequently assessed directly [39].

In terms of pharmacological interventions, our results mirror those of Burchardi and Schneider, who noted the high cost and variable effectiveness of therapies for severe sepsis, cautioning against broad adoption without robust cost–benefit justification [40]. The ICERs reported in our study for agents such as drotrecogin alfa and angiotensin II reflect this tension between clinical benefit and economic burden.

Importantly, omega-3 parenteral nutrition—a relatively novel intervention evaluated by Pradelli et al. (2020)—was found to be cost-effective in ICU populations, with improvements in sepsis rates and ICU stay duration [41]. This highlights the evolving landscape of supportive care therapies, where nutritional or adjunctive strategies may offer favorable value.

### 4.2. Preventive and Quality-Improvement Interventions: High Value at Low Cost

One of the most consistent findings across included studies was the economic dominance of preventive measures and quality-improvement (QI) programs. Interventions such as hand hygiene campaigns [35], closed IV infusion systems [33], multilayered silicone foam dressings [34], and broader QI frameworks [31] were found to be either cost-saving or associated with low incremental cost-effectiveness ratios (ICERs). These results resonate with previous literature demonstrating the economic and clinical value of basic preventive strategies in reducing healthcare-associated infections and pressure injuries in ICU settings [42,43].

Importantly, such interventions often target system-level processes and benefit from scalability and sustainability, characteristics that may explain their favorable economic profiles. From a policy perspective, these findings advocate for prioritizing implementation of low-cost, high-impact preventive programs, especially in low- and middle-income countries where ICU resources are constrained [44].

### 4.3. Interpretation of Cost-Effectiveness Trends

Across the included studies, preventive interventions were more consistently cost-saving or dominant. These findings support health system investment in scalable quality improvement strategies—particularly in resource-constrained settings. By contrast, high-cost pharmacologic agents and supportive technologies (e.g., mechanical ventilation, pulmonary artery catheters) often yielded higher ICERs, sometimes exceeding conventional thresholds for cost-effectiveness. While life-years gained were frequently reported as the primary measure of effectiveness, this metric may not fully capture quality-of-life adjustments reflected in QALYs, underscoring that survival gains may not always translate into improved quality-adjusted outcomes.

The variability in ICERs across interventions suggests the need for context-sensitive evaluations, particularly given differences in ICU organization, baseline mortality risk, and national willingness-to-pay thresholds. Our findings confirm those of Dos Santos et al., who concluded that the cost-effectiveness of ICU antimicrobial therapies depends heavily on clinical context and drug resistance prevalence [45].

### 4.4. Pharmacologic and Life-Support Interventions: Substantial Variation in Economic Value

In contrast to preventive measures, pharmacological interventions, and advanced life-support therapies demonstrated a wide spectrum of cost-effectiveness. For example, drotrecogin alfa (activated) produced ICERs ranging from $19,664 [23] to $78,719 per QALY [24], while angiotensin II [36] and polymyxin B hemoperfusion [22] were more economically favorable. On the other hand, mechanical ventilation [28] and probiotics [29] resulted in extremely high ICERs, exceeding commonly accepted willingness-to-pay (WTP) thresholds.

These discrepancies reflect multiple underlying factors, including differences in study design, model assumptions, healthcare system costs, and population characteristics. Notably, some interventions, such as hydrocortisone [27] or sepsis management protocols [26], were shown to be dominant—improving outcomes while reducing costs. Such results echo findings from trials like the ADRENAL [46] and PROWESS [47], which have examined these therapies under varying cost and care structures.

Nonetheless, the variability of results among pharmacologic interventions cautions against generalizing economic favorability across settings. Contextual adaptation and localized cost modeling remain essential.

### 4.5. Meta-Analytic Evidence

The meta-analysis indicates a positive overall trend towards cost-effectiveness of ICU interventions, as reflected in the pooled INB estimate. Although variability across studies was evident, the direction of effect supports the economic value of several strategies in critical care. The funnel plot did not show strong evidence of systematic bias, though the limited number of studies reduces the certainty of this assessment. Overall, the findings reinforce the potential of ICU interventions to deliver both clinical and economic benefits, while highlighting the need for continued generation of high-quality trial-based evaluations.

### 4.6. Methodological Quality and Reporting Gaps

The methodological quality of included studies, assessed using the CHEERS 2022 checklist, was generally high, with an average score of 79%. However, several recurrent limitations were noted. Most notably, the absence of predefined economic analysis plans and failure to report distributional effects or patient engagement undermines transparency and reproducibility. Furthermore, critical modeling assumptions were frequently underreported, limiting interpretability.

Only a minority of studies included lifetime horizons or adopted a societal perspective, despite recommendations from major guidelines [48]. Short time horizons, such as the 12-day window in El Genedy et al. (2020), may underestimate downstream costs or benefits, particularly in chronic critical illness or survivors of sepsis [34].

Moreover, the lack of stakeholder engagement across almost all studies is concerning, given the growing emphasis on patient-centered economic evaluations and health equity [49].

### 4.7. Implications for Practice and Policy

This review highlights the potential of cost-effectiveness evidence to guide ICU practice reform. Interventions such as hand hygiene, protocolized sepsis care, and prevention of device-associated infections demonstrated favorable or dominant economic profiles and should be prioritized for implementation, especially in settings with constrained ICU capacity or high infection burden.

Interventions identified as economically dominant shared several characteristics: they targeted preventable complications or system-level processes, involved relatively low implementation costs, and produced sustained reductions in infection rates, resource utilization, or length of stay. Unlike pharmacologic therapies, these interventions deliver diffuse benefits across patient populations and time, contributing to both cost savings and improved outcomes. The most economically dominant interventions—such as infection prevention, hand hygiene, and protocol adherence—are low-cost, system-level measures that receive little commercial attention yet deliver substantial health and economic benefits.

However, care must be taken to contextualize economic data to local practice environments. Country-specific threshold values, health system financing models, and treatment availability influence both the costs and effectiveness of ICU interventions.

An additional consideration concerns the transferability of cost-effectiveness results across healthcare systems. ICU organization, resource availability, and unit costs vary widely between countries and even between hospitals, directly influencing both cost and effectiveness components of economic evaluations. As such, evidence generated in one context—particularly high-income settings—may not be fully applicable to regions with different health financing models, labor costs, and technology availability. The wide variation in reported costs, particularly in U.S. studies, largely reflects structural differences in health system financing, labor costs, and resource intensity. These factors strongly influence cost-per-outcome estimates and contribute to the heterogeneity observed in cost-effectiveness results, limiting the comparability of pooled estimates across settings. These contextual differences underscore the need for country-specific analyses and standardized reporting frameworks to enhance external validity.

### 4.8. Limitations and Future Research

Our synthesis is limited by heterogeneity in study populations, comparators, and outcome measures. Additionally, many included evaluations predate recent changes in ICU care (e.g., COVID-19-related protocols, bundled payments), potentially limiting applicability. Finally, while ICERs remain standard, their use without net benefit frameworks may obscure true value under uncertainty.

It should also be noted that the definition of “standard care” varied across included studies, reflecting local clinical protocols and resource availability. This variability likely contributed to some of the observed heterogeneity in incremental cost-effectiveness ratios and incremental net benefits.

Additionally, the reconstruction of missing variance estimates, while based on validated approaches, introduces potential measurement error and uncertainty. The limited reporting of variance and uncertainty measures in several primary studies may have reduced the precision of estimated variances, although the general trend of pooled incremental net benefits remained consistent across studies.

Finally, although the CHEERS 2022 checklist ensured a structured and transparent assessment of reporting quality, it is not designed to capture methodological rigor or risk of bias. Consequently, the use of CHEERS as the sole appraisal tool may have limited the depth of quality assessment in this review.

Future research should emphasize multi-country evaluations using real-world data, greater use of QALYs and patient-reported outcomes, and full probabilistic modeling to assess uncertainty.

## 5. Conclusions

This systematic review and meta-analysis provide a comprehensive synthesis of trial-based economic evaluations in adult intensive care settings, addressing the persistent challenges of escalating costs, limited consolidated evidence, and substantial methodological heterogeneity that characterize research in this field. The analysis demonstrates wide variability in cost-effectiveness across interventions, clinical contexts, and healthcare systems, reflecting differences in design, analytic perspective, and resource availability.

Preventive strategies and quality-improvement initiatives—such as hand hygiene programs, closed infusion systems, and protocolized care pathways—were consistently found to be economically dominant, delivering improved patient outcomes at lower or comparable costs. In contrast, pharmacological and advanced life-support interventions exhibited highly variable economic profiles, ranging from cost-effective to prohibitively expensive depending on context and study design.

The pooled analysis of incremental net benefit suggests a trend toward positive economic value of ICU interventions at standard willingness-to-pay thresholds; however, substantial heterogeneity underscores the need for cautious interpretation and context-specific appraisal. Methodological inconsistencies, particularly in planning transparency, stakeholder engagement, and perspective selection, further limit cross-study comparability.

By systematically consolidating trial-based cost-effectiveness evidence across adult ICU populations, this review addresses a major gap in the existing literature, which has previously been dominated by model-based or intervention-specific analyses. It is, to our knowledge, the first meta-analysis to quantify pooled incremental net benefits from empirical economic evaluations in critical care. Therefore, it provides a unified and policy-relevant synthesis that enhances understanding of where economic value can be achieved in ICU practice. Beyond summarizing existing data, this review advances the evidence base by identifying methodological shortcomings, highlighting areas where reporting and standardization are most needed, and outlining directions for future research and policy development.

Policymakers and healthcare providers should consider the economic value of ICU-based strategies in conjunction with clinical effectiveness, local resource constraints, and system-level priorities. Future economic evaluations should embrace comprehensive reporting standards, include equity and stakeholder dimensions, and leverage real-world data to inform context-sensitive, evidence-based critical care policies.

## Figures and Tables

**Table 2:** Primary Cost-Effectiveness Outcomes.

Study	Country	Primary Outcome ICER
Berto, 2011 [22]	Italy	Cost per LYG: 6904
Tarricone, 2010 [33]	Italy	Cost per CLABSI prevented: Dominant
Heyland, 1998 [30]	Canada	Cost per LYG: 7176 Cost per Life Saved: 107,592
Manns, 2002 [24]	Canada	Cost per LYG: 47,231
Busse, 2020 [36]	9 countries (North America, Australasia, and Europe)	Cost per LYG: 10,756
Ersson, 2018 [31]	Sweden	Cost per LYG: Dominant
Lau, 2022 [29]	3 countries (Canada/USA/Saudi Arabia)	Cost per Life Saved: 476,499
Mayer, 2000 [28]	USA	Cost per LYG: 69,346
Dhainaut, 2007 [25]	France	Cost per LYG: 36,042
El Genedy, 2020 [34]	Germany	Cost per PU avoided: 3091
Thu, 2015 [35]	Vietnam	Cost per HAI prevented: Dominant
Riou Franca, 2006 [23]	France	Cost per LYG: 19,664
Thompson, 2022 [27]	New Zealand	Cost per LYG: Dominant
Stevens, 2005 [32]	Great Britain	Cost per Life Saved: 51,664
Assuncao, 2014 [26]	Brazil	Cost per LYG: Dominant Cost per Life Saved: Dominant

**Table 3:** Secondary Cost-Effectiveness Outcome (Cost per QALY).

Study	Country	ICER
Manns, 2002 [24]	Canada	Cost per QALY: 78,719
Busse, 2020 [36]	9 countries (North America, Australasia, and Europe)	Cost per QALY: 15,700
Ersson, 2018 [31]	Sweden	Cost per QALY: Dominant
Mayer, 2000 [28]	USA	Cost per QALY: 321,280
Dhainaut, 2007 [25]	France	Cost per QALY: 60,070
RiouFranca, 2006 [23]	France	Cost per QALY 32,772
Thompson, 2022 [27]	New Zealand	Cost per QALY Dominated
Stevens, 2005 [32]	Great Britain	Cost per QALY: 7059

**Table 4:** Descriptive of the Incremental Net Benefit.

Study	INB ($)	95% CI
Berto, 2011 [22]	102,747	−53,320 to 258,814
Heyland, 1998 [30]	151,557	90,422 to 212,691
Manns, 2002 [24]	−6548	−14,172 to 1076
Busse, 2020 [36]	18,437	6549 to 30,324
Mayer, 2000 [28]	−67,675	−116,687 to −18,662
Dhainaut, 2007 [25]	−3323	−29,918 to 23,272
RiouFranca, 2006 [23]	6640	−588 to 13,868
Thompson, 2022 [27]	42,800	27,825 to 57,774

## Abbreviations

The following abbreviations are used in this manuscript:

ICU	Intensive Care Unit
CEA	Cost-Effectiveness Analysis
ICER	Incremental Cost-Effectiveness Ratio
LYG	Life-Years Gained
QALY	Quality-Adjusted Life Year
INB	Incremental Net Benefit
CHEERS	Consolidated Health Economic Evaluation Reporting Standards
CI	Confidence Interval
CEAC	Cost-Effectiveness Acceptability Curve
ISPOR	International Society for Pharmacoeconomics and Outcomes Research
PRISMA	Preferred Reporting Items for Systematic Reviews and Meta-Analyses
CRD	Centre for Reviews and Dissemination
USD	United States Dollar
SD	Standard Deviation
SE	Standard Error
WTP	Willingness to Pay

## Appendix A

**Table A1:** CHEERS evaluation.

	Dhainaut, 2007 [25]	Berto, 2011 [22]	Busse, 2020 [36]	Ersson, 2018 [31]	Riou Franca, 2006 [23]	El Genedy, 2020 [34]	Heyland, 1998 [30]	Manns, 2002 [24]	Stevens, 2005 [32]	Tarricone, 2010 [33]	Thu, 2015 [35]	Lau, 2022 [29]	Mayer, 2000 [28]	Thompson, 2022 [27]	Assuncao, 2014 [26]
Title	1	1	1	0	1	1	1	1	1	1	1	0.5	1	1	1
Abstract	1	1	1	1	1	1	1	0.5	1	1	1	1	0.5	1	0.5
Background and objectives	1	1	1	1	1	1	1	1	1	1	1	1	1	1	1
Health economic analysis plan	0	0	0	0	0	0	0	0	0	0	0	1	0	1	0
Study population	1	1	1	1	1	1	1	1	1	1	1	1	0.5	1	1
Setting and Location	1	1	1	1	1	1	1	1	1	1	1	1	0.5	1	1
Comparators	1	1	1	1	1	1	1	1	1	1	1	1	1	1	1
Perspective	1	1	1	1	0	1	1	1	1	1	0	1	1	1	0
Time Horizon	0.5	1	1	0.5	1	1	1	1	0.5	1	0	1	0.5	1	0
Discount Rate	0.5	1	1	1	1	NA	NA	1	1	NA	NA	1	1	0	0
Selection of Outcomes	1	1	1	1	1	1	1	1	1	1	1	1	1	1	1
Measurement of Outcomes	1	1	1	1	1	1	1	1	1	1	1	1	1	1	1
Valuation of outcomes	1	1	1	1	1	0	0	1	1	1	0	1	1	1	0.5
Measurement and valuation of resources and costs	1	1	1	1	1	1	1	1	1	1	1	1	1	1	0.5
Currency, price date, and conversion	1	1	1	1	1	0.5	1	1	1	0.5	1	1	1	1	1
Rationale and description of the model	NA	1	0.5	N	0	1	1	0.5	N	1	N	N	0.5	NA	NA
Analytics and assumptions	1	1	1	N	1	0.5	1	1	1	1	1	1	1	1	1
Characterizing Heterogeneity	1	N	0	N	1	0.5	0	1	0	0	0	1	0.5	1	0
Characterizing distributional effects	0	N	0	N	0	0	0	0	0	0	0	0	0	0	0
Characterizing uncertainty	1	1	1	1	1	1	1	1	1	0	1	1	1	1	0
Approach to engagement	0	0	0	0	0	0	0	0	0	0	0	1	0	0	0
Study parameters	1	1	1	1	1	1	1	1	0.5	0.5	1	1	1	0.5	0.5
Summary of main results	1	1	1	1	1	1	1	1	1	0.5	1	1	1	1	1
Effect of uncertainty	1	1	1	1	1	1	0.5	1	1	0	0.5	1	0.5	1	0
Effect of engagement	NA	NA	NA	NA	0	NA	NA	NA	NA	0	NA	NA	NA	NA	NA
Study findings	1	1	1	1	1	1	1	1	1	1	1	1	1	1	1
Source of funding	1	1	1	1	0.5	1	0	1	1	1	1	1	1	1	0
Conflicts of interest	1	1	1	1	0	1	0	0	1	1	1	1	0	0	1
% Score	85%	92%	83%	85%	73%	79%	71%	81%	81%	69%	70%	94%	72%	83%	54%

NA = not applicable.
